# Importance of hematological parameters for micro- and macrovascular outcomes in patients with type 2 diabetes: the Rio de Janeiro type 2 diabetes cohort study

**DOI:** 10.1186/s12933-021-01324-4

**Published:** 2021-07-06

**Authors:** Claudia R. L. Cardoso, Nathalie C. Leite, Gil F. Salles

**Affiliations:** Department of Internal Medicine, University Hospital Clementino Fraga Filho, School of Medicine; Universidade Federal Do Rio de Janeiro, Rua Croton, 72, Rio de Janeiro, Jacarepagua 22750-240 Brazil

**Keywords:** Hematologic parameters, Cardiovascular outcomes, Cohort study, Microvascular complications, Mortality, Type 2 diabetes

## Abstract

**Background:**

The prognostic importance of several hematological parameters has been scarcely investigated in type 2 diabetes. So, we aimed to evaluate their prognostic importance for development of complications in a cohort of type 2 diabetes.

**Methods:**

In a prospective study, 689 individuals with type 2 diabetes had blood red cell, platelet and leukocyte parameters obtained at baseline. Multivariate Cox analyses examined the associations between several hematological parameters (including neutrophyl-to-lymphocyte, lymphocyte-to-monocyte, platelet-to-lymphocyte, and monocyte-to-HDL ratios) and the occurrence of microvascular (retina, renal and peripheral neuropathy) and cardiovascular complications (total cardiovascular events [CVEs], and major adverse CVEs [MACEs]), and all-cause and cardiovascular mortality. Improvements in risk discrimination were assessed by C-statistics and Integrated Discrimination Improvement (IDI) index.

**Results:**

During a median follow-up of 10.5 years, 212 patients had a CVE (174 MACEs), 264 patients died (131 cardiovascular deaths); 206 had a renal, 161 a retinopathy and 179 patients had a neuropathy outcome. In multivariate-adjusted analyses, the lymphocytes count and lymphocyte-to-monocyte ratio were protective (hazard ratios [HRs]: 0.77 and 0.72, respectively), whereas the neutrophyl-to-lymphocyte and platelet-to-lymphocyte ratios were associated with increased risks (HRs: 1.19 and 1.17) for all-cause mortality. For cardiovascular mortality, the monocytes count, the neutrophyl-to-lymphocyte and monocyte-to-HDL ratios were associated with increased risks and the lymphocyte-to-monocyte ratio was protective. Higher lymphocyte-to-monocyte ratio was protective for renal failure outcome. However, none of them improved risk discrimination.

**Conclusions:**

Low lymphocytes count and leukocyte ratios that mainly included lymphocytes were predictors of macrovascular complications and mortality in individuals with type 2 diabetes. However, they did not improve risk prediction over traditional risk factors.

## Background

Type 2 diabetes is a major health problem worldwide. The chronic micro- and macrovascular complications that increase with diabetes severity and duration are responsible for a great burden of disease-related morbidity and mortality. Systemic low-grade chronic inflammation has long been recognized as a central biological mainstay of diabetes and growing evidences suggest that inflammation plays an important role in diabetes-related complications [1, 2]. Indeed, atherosclerosis, which underlies the macrovascular complications, is currently regarded as a chronic inflammatory disease [1]. Inflammatory cells contribute to atherosclerotic lesion initiation, progression and its disruption that cause cardiovascular events [3]. Further, there are also evidences that inflammation may play a key role in occurrence of microvascular complications [2, 4–8].

In special, blood leukocytes are important in the course of vascular wall deterioration in individuals with diabetes, being involved in the progression of atherosclerosis and in destabilization and disrupt of plaque, culminating in atherothrombotic events [9, 10]. Increasing concern has been demanded to leukocytes subtypes and its relations. Indeed, the neutrophyl-to-lymphocyte and the lymphocyte-to-monocyte ratios, and additionally other relations such as the platelet-to-lymphocyte, monocyte-to-HDL-cholesterol and also other hematological parameters such as the red blood cell distribution width (RDW), have been investigated as potential risk markers in several clinical conditions [3, 6–8, 11–19]. These are inexpensive and easily-obtained measures to investigate inflammatory parameters and potential risk markers. The neutrophyl-to-lymphocyte ratio (NLR) is the most widely investigated and links two different pathways that initiate the inflammatory response. Neutrophyls are intimately associated with proceeding inflammation and lymphocytes reflect the immune regulatory response pathway [20, 21].

Several studies investigated the relationships between hematological parameters and the presence of degenerative complications in type 2 diabetes [3, 6–8, 11, 13–15, 17, 18], but few explored their prognostic importance for adverse outcomes in longitudinal analyses [11, 22, 23]. Otherwise, these previous studies were mainly retrospective [11, 22] and focused on specific hematological parameters, such as the NLR [11, 22] and monocytes count [23], and in specific outcomes, such as cardiovascular events [11], renal function deterioration [22] and all-cause mortality [23]. The prognostic importance of other hematological parameters, such as the lymphocyte-to-monocyte, platelet-to-lymphocyte, or monocyte-to-HDL ratios, has never been investigated in type 2 diabetes; only simple associations were examined in cross-sectional analyses [6, 15, 17, 18, 24]. Moreover, none of the previous longitudinal studies [11, 22, 23] evaluated whether any of the hematological parameters was able to improve risk discrimination for the specific analyzed outcome. Hence, a comprehensive longitudinal analysis on the prognostic importance of several hematological parameters for macro- and microvascular complications outcomes in diabetes has never been performed yet, and it is needed.

Therefore, we intended to investigate in a prospective cohort of middle-aged type 2 diabetic individuals the prognostic value of several hematological parameters for future occurrence of macro- and microvascular complications and mortality, and to explore if they improve risk discrimination over and beyond traditional risk markers.

## Methods

### Patients and baseline procedures

This prospective study included 689 individuals with type 2 diabetes from the Rio de Janeiro Type 2 Diabetes (RIO-T2D) Cohort Study, enrolled between August 2004 and December 2008 and followed-up until June 2019 in the diabetes outpatient clinic of our tertiary-care University Hospital. All participants gave written informed consent, and the local Ethics Committee had previously approved the study protocol. The characteristics of this cohort, the baseline procedures and the diagnostic definitions have been described previously [25–29]. In summary, inclusion criteria were all adult type 2 diabetic individual up to 80 years old with either any microvascular (retinopathy, nephropathy or neuropathy) or macrovascular (coronary, cerebrovascular or peripheral artery disease) complication, or with at least two other modifiable cardiovascular risk factors. Exclusion criteria were morbid obesity (body mass index ≥ 40 kg/m^2^), advanced renal failure (serum creatinine > 180 μmol/L or estimated glomerular filtration rate < 30 ml/min/1.73 m^2^) or the presence of any serious concomitant disease limiting life expectancy. All participants were submitted to a standard baseline protocol that included a thorough clinical-laboratory evaluation. Diagnostic criteria for diabetic chronic complications were detailed previously [25–29]. In brief, coronary heart disease was diagnosed by clinical, electrocardiographic criteria, or by positive ischemic stress tests. Cerebrovascular disease was diagnosed by history and physical examination, and peripheral arterial disease by an ankle-brachial index < 0.9. The diagnosis of nephropathy needed at least two albuminurias ≥ 30 mg/24 h or proteinurias ≥ 0.5 g/24 h or confirmed reduction of glomerular filtration rate (eGFR ≤ 60 ml/min/1.73 m^2^, estimated by the CKD-EPI equation, or serum creatinine > 130 μmol/L). Peripheral neuropathy was determined by clinical examination (knee and ankle reflex activities, feet sensation with the Semmes–Weinstein monofilament, vibration with a 128-Hz tuning fork, pinprick and temperature sensations) and neuropathic symptoms were assessed by a standard validated questionnaire [27]. Clinic blood pressure (BP) was measured three times using a digital oscillometric BP monitor (HEM-907XL, Omron Healthcare, Kyoto, Japan) with a suitable sized cuff on two occasions two weeks apart at study entry [25]. The first measure of each visit was discarded and BP considered was the mean between the last two readings of each visit. Arterial hypertension was diagnosed if mean systolic (SBP) ≥ 140 mmHg or diastolic BP (DBP) ≥ 90 mmHg or if anti-hypertensive drugs had been prescribed. Laboratory evaluation included hemogram, fasting glycemia, glycated hemoglobin (HbA_1c_), serum creatinine and lipids. Albuminuria and proteinuria were evaluated in two non-consecutive sterile 24-h urine collections.

### Assessment of hematological parameters

Fasting blood samples were collected for performing all laboratory exams in the Central Laboratory of our University Hospital. A complete blood count was performed with an automated hematology analyzer (Coulter LH-750 Analyzer, Beckman Coulter, USA), and it included hemoglobin concentration and red blood cell distribution width (RDW), total leukocytes, neutrophyls, lymphocytes, monocytes and platelets counts. The neutrophyl-to-lymphocyte, lymphocyte-to-monocyte, platelet-to-lymphocyte and monocyte-to-HDL ratios were calculated using the complete blood count measures. No participant had any signs or symptoms of any acute clinical condition, including bacterial or viral infections, prior to blood sampling. All individuals who had abnormally low or high leucocytes count had their exam repeated after a month to confirm the results; if they were discordant, it was considered the one with normal values, and if they were concordant, it was considered the first collected.

### Follow-up and outcomes assessment

The patients were followed-up regularly at least 3–4 times a year until June 2019 under standardized treatment. The observation period for each patient was the number of months from the date of the first clinical examination to the date of the last clinical visit until June 2019 or the date of the first endpoint, whichever came first. The primary outcomes were the occurrence of any macrovascular or microvascular events. Macrovascular outcomes were total cardiovascular events (CVEs: fatal or non-fatal myocardial infarctions [MIs], sudden cardiac deaths, new-onset heart failure, death from progressive heart failure, any myocardial revascularization procedure, fatal or non-fatal strokes, any aortic or lower limb revascularization procedure, any amputation above the ankle, and deaths from aortic or peripheral arterial disease), major adverse cardiovascular events (MACEs: non-fatal MIs and strokes plus cardiovascular deaths), and all-cause and cardiovascular mortalities [25, 26]. Microvascular outcomes were retinopathy development or worsening [28], renal outcomes [29] (new microalbuminuria development, and renal function deterioration [defined as doubling of serum creatinine or end-stage renal failure needing dialysis or death from renal failure], and a composite of them), and peripheral neuropathy development or worsening [27]. Retinopathy and renal outcomes were evaluated by annual examinations [28, 29], whereas peripheral neuropathy was evaluated on two serial specific examinations performed after a median of 6 and 10 years from the baseline examination [27, 30].

### Statistical analyses

Continuous data were described as means (SD) or as medians (interquartile range). For initial exploratory analyses, patients were categorized into tertiles of the NLR (because this is the most widely-used ratio) and baseline characteristics compared by ANOVA, Kruskal–Wallis or χ^2^ tests, when appropriate. Kaplan–Meier curves of cumulative endpoints incidence during follow-up, compared by log-rank tests, were used for assessing different incidences of outcomes among tertile subgroups. For assessing the prognostic value for each macrovascular and microvascular outcome, except for peripheral neuropathy, a time-to-event Cox analysis was undertaken with progressively increasing statistical adjustments for potential confounding. Model 1 was only adjusted for age and sex; and Model 2 was further adjusted for other potential confounders (diabetes duration, body mass index [BMI], smoking status, physical inactivity, office SBP, number of anti-hypertensive drugs in use, presence of micro- and macrovascular complications at baseline, baseline HbA_1c_, HDL- and LDL-cholesterol levels, and use of insulin, statins and aspirin). These results were presented as hazard ratios (HRs) with their 95% confidence intervals (CIs). For peripheral neuropathy analyses, a multiple logistic regression was used with the same progressively increasing statistical adjustments, except that height (instead of BMI) and the time-interval between the baseline and the other 2 neuropathy evaluations were included as adjusting covariates. These results were reported as odds ratios (ORs) with their respective 95% CIs. Hematological parameters were examined both as continuous variables, with HRs and ORs estimated for increments of 1-SD to allow comparisons among them; and also categorized into tertiles, with HRs and ORs estimated for the highest tertile subgroup in relation to the reference lowest tertile subgroup. If any of the hematological parameters was demonstrated to be a significant predictor of any of the outcomes, then the improvement in risk discrimination of adding this parameter over a standard risk factor model (composed by those covariates in Model 2) for this specific outcome was tested by the C-statistic (analogous to the area under ROC curve applied to time-to-event analysis), compared by the method proposed by DeLong [31], and by the Integrated Discrimination Improvement (IDI) index [32, 33]. The IDI is equivalent to the difference in discrimination slopes between models with and without the new variable and its calculation is based on continuous differences in predicted risk in new and old models in individual cases (with outcome) and controls (without outcome). Both the absolute and the relative IDIs were calculated. The relative IDI, reported as a percentage, facilitates the IDI clinical interpretation, and is defined as the increase in the discrimination slope divided by the slope of the standard risk model [32, 33]. All statistics were performed with SPSS version 19.0 (SPSS Inc, Chicago, IL, USA) and R version 3.4.1 (R Foundation for Statistical Computing, Vienna, Austria); and a 2-tailed probability value < 0.05 was considered significant.

## Results

Table 1 outlines the baseline characteristics of all the 689 patients included in the study and of those divided into tertiles of NLR. Individuals with higher NLR were leaner, and more frequently smokers than those with lower ratios. Expectedly, they had higher leukocyte and neutrophyl counts and lower lymphocytes than those with lower ratios. They also had higher monocyte counts and higher platelet-to-lymphocyte and lower lymphocyte-to-monocyte ratios. There were no differences in the prevalences of macro- or microvascular complications at baseline, as well as in metabolic and BP control.

**Table 1:** Characteristics of all diabetic patients and divided into tertiles of the neutrophyl-to-lymphocyte ratio

Characteristics	All patients (n = 689)	1st tertile < 1.6 (n = 229)	2nd tertile ≥ 1.6 to < 2.2 (n = 230)	3rd tertile ≥ 2.2 (n = 230)
Age (years)	60.0 (9.6)	60.0 (9.4)	59.0 (9.4)	61.0 (9.8)
Male sex (%)	39.2	34.5	37.8	45.2
BMI (kg/m^2^)	29.7 (4.8)	30.1 (4.8)	30.1 (4.9)	29.0 (4.8)‡
Smoking, current/past (%)	45.1	43.2	39.7	52.2‡
Physical activity (%)	22.2	24.0	19.2	23.5
Diabetes duration (years)	8 (3–15)	7 (3–14)	7 (3–14.5)	9 (4–17)
Chronic diabetic complications (%)				
Cerebrovascular disease	9.2	8.7	11.4	7.4
Coronary artery disease	15.1	14.8	11.8	18.7
Peripheral artery disease	16.9	16.6	14.0	20.1
Retinopathy	32.3	31.8	31.5	33.6
Nephropathy	31.6	28.1	29.3	37.4
Peripheral neuropathy	29.1	30.1	25.1	31.9
Cardiovascular autonomic neuropathy	24.7	20.6	26.5	27.0
Diabetes treatment (%)				
Metformin	87.9	88.2	88.6	87.0
Sulfonylureas	43.0	40.6	45.0	43.5
Insulin	48.1	48.0	48.9	47.4
Aspirin	89.6	88.2	88.3	92.2
Dyslipidemia (%)	87.1	86.5	86.0	88.7
Statins use (%)	77.0	75.0	73.4	82.5‡
Arterial hypertension (%)	86.6	86.5	86.0	88.7
Number of anti-hypertensive drugs	3 (1–3)	3 (1–3)	2 (1–3)	3 (2–4)
Blood pressures (mmHg)				
Mean clinic SBP	140 (19)	140 (19)	140 (20)	141 (19)
Mean clinic DBP	79 (11)	79 (11)	80 (11)	79 (10)
Hematological parameters				
Haemoglobin (g/dl)	13.7 (1.5)	13.6 (1.5)	13.7 (1.5)	13.6 (1.6)
RDW (%)	13.1 (1.3)	13.0 (1.5)	13.0 (1.3)	13.2 (1.4)
Leukocytes (× 10^3^ cells/mm^3^)	7.4 (2.1)	6.7 (2.0)	7.3 (2.0)†	8.1 (2.1)*
Neutrophyls (× 10^3^ cells/mm^3^)	4.3 (1.6)	3.2 (1.1)	4.2 (1.1)*	5.4 (1.6)*
Lymphocytes (× 10^3^ cells/mm^3^)	2.2 (0.8)	2.7 (0.9)	2.3 (0.7)*	1.8 (0.5)*
Monocytes (cells/mm^3^)	500 (185)	473 (183)	497 (171)	528 (197)†
Platelets (× 10^3^ cells/mm^3^)	244 (74)	250 (83)	243 (65)	241 (74)
Lymphocyte-to-monocyte ratio	5.0 (2.4)	6.4 (2.8)	4.9 (1.7)*	3.8 (1.8)*
Platelet-to-lymphocyte ratio	118.2 (46.3)	98.4 (35.8)	113.5 (35.7)*	142.6 (52.4)*
Monocyte-to-HDL ratio	12.7 (6.3)	12.0 (5.9)	12.7 (6.5)	13.3 (6.5)
Laboratory variables				
Mean HbA_1c_ (%)	7.7 (1.6)	7.7 (1.6)	7.6 (1.5)	7.7 (1.6)
(mmol/mol)	61 (11.6)	61 (11.6)	60 (10.5)	61 (11.6)
Mean triacylglycerol (mmol/L)	1.55 (1.08–2.22)	1.60 (1.14–2.29)	1.59 (1.12–2.30)	1.43 (1.00–2.07)
Mean HDL-cholesterol (mmol/L)	1.14 (0.44)	1.14 (0.28)	1.11 (0.28)	1.16 (0.65)
Mean LDL-cholesterol (mmol/L)	2.77 (0.85)	2.84 (0.83)	2.79 (0.88)	2.74 (0.85)
C-reactive protein (CRP) mg/L	2.9 (1.2–6.2)	2.7 (0.9–6.1)	3.1 (1.5–6.3)	2.8 (1.3–6.5)
Glomerular filtration rate (ml/min/1.73 m^2^)	81 (20)	82 (19)	83 (21)	79 (21)
Albuminuria (mg/24 h)	13 (7–41)	14 (7–32)	13 (7–39)	15 (7–52)
Macrovascular outcomes^a^				
Total CV events	212 (35.2)	62 (29.9)	61 (29.9)	89 (46.7)†
Major CV events	174 (27.8)	56 (26.5)	45 (21.2)	73 (36.3)†
Cardiovascular mortality	131 (20.1)	44 (19.9)	30 (13.6)	57 (27.3)†
All-cause mortality	264 (40.5)	80 (36.2)	78 (35.2)	106 (50.7)†
Microvascular outcomes^b^				
Retinopathy (incident/worsening) (n = 551)	161 (50.7)	51 (49.5)	56 (53.2)	54 (49.4)
Renal composite	206 (37.7)	67 (36.1)	63 (33.3)	76 (44.4)
Microalbuminuria (incident)	127 (25.6)	43 (25.4)	35 (20.4)	49 (31.4)
Renal failure	104 (17.0)	29 (14.0)	34 (16.4)	41 (21.0)
Peripheral neuropathy (incident/worsening) (n = 525)	179 (34.1%)	51 (28.5%)	65 (36.9%)	63 (37.1%)

Values are proportions, and means (standard deviations) or medians (interquartile range). *BMI* body mass index, *SBP* systolic blood pressure, *DBP* diastolic blood pressure, *RDW* red cell distribution width, *HDL* high-density lipoprotein, *LDL* low-density lipoprotein, *HbA*_*1c*_ glycated hemoglobin, *CV* cardiovascular

^a^Values are absolute numbers (incidence rate per 1000 patient-years of follow-up)

^b^Values are absolute numbers (incidence rate per 1000 patient-years of follow-up), except for peripheral neuropathy that are absolute numbers (proportions)

‡p < 0.05; †p < 0.01; *p < 0.001 (after Bonferroni’s correction) for comparisons with the reference 1st tertile subgroup

### Endpoints incidence during follow-up

**Fig. 1:**
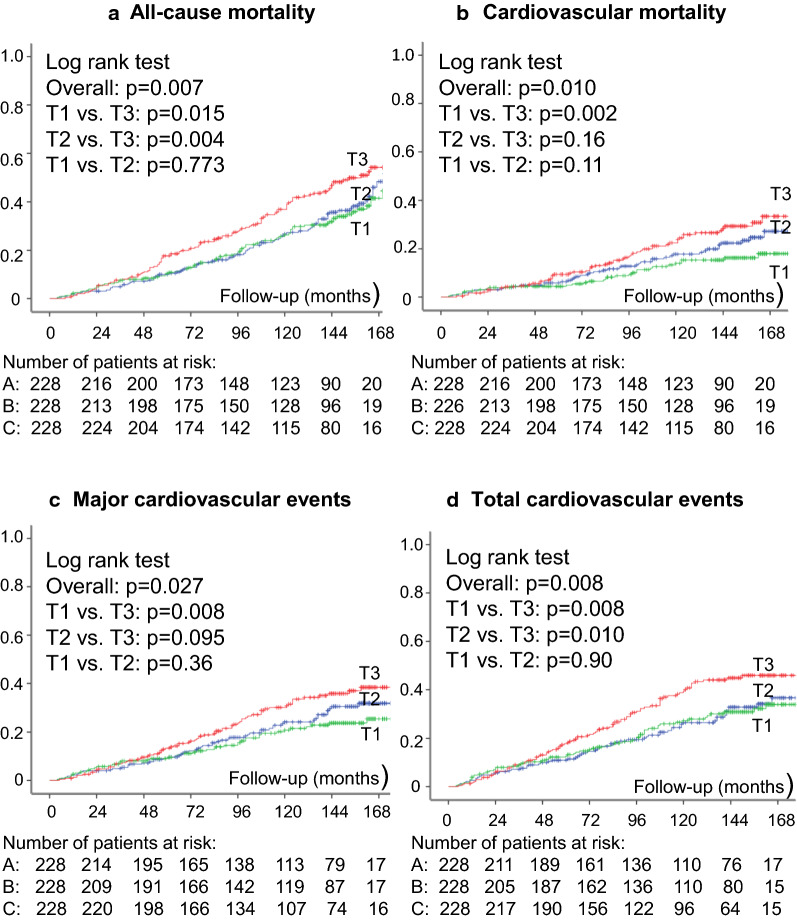
Kaplan–Meier estimation curves of cumulative incidences of adverse outcomes all-cause mortality (**a**); cardiovascular mortality (**b**); major adverse cardiovascular events (**c**); and total cardiovascular events (**d**), for patients divided into tertiles (T1, lowest; T2, middle; T3 highest) of the neutrophyl-to-lymphocyte ratio

Over a median follow-up of 10.5 years (IQR: 6.3–13 years, maximum 16.2 years), 212 patients had a CVE (174 MACEs); and 264 patients died, 131 from cardiovascular diseases. One-hundred and sixty-one newly-developed or worsened diabetic retinopathy, 206 achieved the renal composite outcome (127 newly developed microalbuminuria and 104 deteriorated renal function), and 179 newly-developed or worsened peripheral neuropathy. Table 1 (bottom) shows the incidence rates of endpoints in participants divided into tertiles of the NLR. Individuals in the top tertile subgroup had higher incidences of total CVEs, MACEs, and of all-cause and cardiovascular mortality than those in the lower tertiles. There was no difference in the incidence of any microvascular outcome among tertiles of NLR. Kaplan–Meier curves of cumulative incidence of events over time according to tertiles of NLR (Fig. 1) confirmed the increased incidence of these outcomes in the highest tertile in relation to the middle and lowest tertile subgroups.

### Risks associated with hematological parameters for incident macrovascular complications and mortality

Tables 2 (analyses with continuous variables) and 3 (analyses with variables categorized into tertiles) present the adjusted risks of hematological parameters for occurrence of cardiovascular complications and mortality. In multivariate-adjusted analyses of continuous variables (Table 2), no parameter predicted cardiovascular outcomes, except cardiovascular mortality (monocytes count, and neutrophyl-to-lymphocyte, lymphocyte-to-monocyte, and monocyte-to-HDL ratios). However, none of them improved risk discrimination, as assessed by C-statistics increase or IDI index. The highest relative IDI was a statistically-borderline (0.10 < p-value > 0.05) 15.5% improvement obtained by adding either monocytes or monocyte-to-HDL ratio to the baseline risk model. For all-cause mortality, the lymphocytes count and all the 3 ratios with lymphocytes predicted the outcome, but again none of them improved risk discrimination. In multivariate-adjusted categorical analyses (Table 3), individuals in the highest tertile subgroup of lymphocytes count had significantly lower risks of total cardiovascular events and mortality, and those in the highest tertile of neutrophyl-to-lymphocyte and platelet-to-lymphocyte ratios had higher risks of cardiovascular complications and mortality than those in the lowest tertile subgroup. However, none of them was able to improve risk discrimination for any of the outcomes. In categorical analyses, the highest relative IDI was a non-significant 8.2% improvement for cardiovascular mortality obtained by adding the platelet-to-lymphocyte ratio.

**Table 2:** Results of Cox survival analyses for the excess risks associated with continuous hematological parameters (increments of 1-SD) for the occurrence of future cardiovascular complications and all-cause mortality

Outcomes Hematological parameters	Model 1 HR (95% CI)	Model 2 HR (95% CI)	C-statistic^a^ (AUC)	Relative IDI^a^ (%)
Total CV events (n = 212)				
Haemoglobin	0.93 (0.80–1.08)	0.98 (0.84–1.14)	–	–
RDW	1.04 (0.91–1.19)	1.05 (0.92–1.21)	–	–
Leukocytes	1.19 (1.04–1.37)‡	1.07 (0.93–1.24)	–	–
Neutrophyls	1.19 (1.04–1.36)‡	1.10 (0.96–1.26)	–	–
Lymphocytes	0.99 (0.84–1.17)	0.96 (0.80–1.14)	–	–
Monocytes	1.18 (1.04–1.35)‡	1.10 (0.95–1.26)	–	–
Platelets	1.14 (0.99–1.31)	1.11 (0.96–1.29)	–	–
Neutrophyl-to-lymphocyte ratio	1.15 (1.02–1.30)‡	1.10 (0.97–1.24)	–	–
Lymphocyte-to-monocyte ratio	0.85 (0.72–1.01)	0.89 (0.75–1.05)	–	–
Platelet-to-lymphocyte ratio	1.07 (0.94–1.23)	1.08 (0.94–1.25)	–	–
Monocyte-to-HDL ratio	1.20 (1.05–1.37)†	1.09 (0.94–1.26)	–	–
Major CV events (n = 174)				
Haemoglobin	0.92 (0.78–1.08)	0.93 (0.79–1.10)	–	–
RDW	1.05 (0.91–1.21)	1.06 (0.92–1.23)	–	–
Leukocytes	1.19 (1.02–1.38)‡	1.07 (0.92–1.25)	–	–
Neutrophyls	1.18 (1.02–1.37)‡	1.10 (0.96–1.27)	–	–
Lymphocytes	0.96 (0.80–1.15)	0.92 (0.76–1.11)	–	–
Monocytes	1.20 (1.04–1.38)‡	1.10 (0.94–1.27)	–	–
Platelets	1.13 (0.98–1.31)	1.12 (0.97–1.29)	–	–
Neutrophyl-to-lymphocyte ratio	1.17 (1.03–1.33)‡	1.13 (0.99–1.29)	–	–
Lymphocyte-to-monocyte ratio	0.86 (0.71–1.03)	0.90 (0.75–1.09)	–	–
Platelet-to-lymphocyte ratio	1.07 (0.93–1.25)	1.10 (0.94–1.28)	–	–
Monocyte-to-HDL ratio	1.24 (1.07–1.43)†	1.12 (0.96–1.31)	–	–
CV mortality (n = 131)			0.716 (0.668–0.765)	
Haemoglobin	0.93 (0.76–1.12)	0.95 (0.78–1.16)	–	–
RDW	1.15 (0.98–1.34)	1.17 (0.99–1.38)	–	–
Leukocytes	1.19 (0.99–1.41)	1.10 (0.91–1.32)	–	–
Neutrophyls	1.21 (1.03–1.44)‡	1.15 (0.97–1.36)	–	–
Lymphocytes	0.87 (0.70–1.08)	0.81 (0.64–1.02)	–	–
Monocytes	1.27 (1.08–1.50)†	1.20 (1.01–1.44)‡	0.724§	15.5%§
Platelets	1.04 (0.87–1.25)	1.02 (0.85–1.22)	–	–
Neutrophyl-to-lymphocyte ratio	1.25 (1.08–1.44)†	1.23 (1.06–1.43)†	0.719§	9.3%§
Lymphocyte-to-monocyte ratio	0.72 (0.57–0.92)†	0.74 (0.58–0.95)‡	0.725§	13.4%§
Platelet-to-lymphocyte ratio	1.08 (0.91–1.29)	1.11 (0.93–1.33)	–	–
Monocyte-to-HDL ratio	1.32 (1.12–1.56)*	1.21 (1.01–1.46)‡	0.722§	15.5%§
All-cause mortality (n = 264)			0.755 (0.717–0.793)	
Haemoglobin	0.86 (0.75–0.98)	0.90 (0.78–1.03)	–	–
RDW	1.08 (0.96–1.21)	1.08 (0.96–1.22)	–	–
Leukocytes	1.10 (0.97–1.25)	1.04 (0.91–1.18)	–	–
Neutrophyls	1.16 (1.03–1.31)‡	1.11 (0.98–1.25)	–	–
Lymphocytes	0.80 (0.68–0.94)†	0.77 (0.65–0.90)†	0.756§	1.6%§
Monocytes	1.20 (1.07–1.35)†	1.13 (0.99–1.28)	–	–
Platelets	1.04 (0.91–1.18)	1.02 (0.89–1.16)	–	–
Neutrophyl-to-lymphocyte ratio	1.23 (1.11–1.37)*	1.19 (1.07–1.33)*	0.757§	2.6%§
Lymphocyte-to-monocyte ratio	0.69 (0.58–0.82)*	0.72 (0.60–0.86)*	0.763§	9.5%§
Platelet-to-lymphocyte ratio	1.17 (1.04–1.31)‡	1.17 (1.03–1.31)‡	0.756§	0.5%§
Monocyte-to-HDL ratio	1.21 (1.07–1.37)†	1.11 (0.97–1.27)	–	–

Values are hazard ratios and 95% confidence intervals estimated for a 1-SD increment in each hematological parameter;*p < 0.001; †p < 0.01;‡p < 0.05. The SDs of the hematological parameters are shown on Table 1

Model 1 was adjusted for age and sex

Model 2 was adjusted for age, sex, diabetes duration, BMI, smoking, physical activity, office systolic BP, number and classes of anti-hypertensive drugs in use, presence of micro- and macrovascular complications at baseline, baseline HbA_1c_ and LDL-cholesterol, and use of insulin, statins and aspirin

*HR* hazard ratio, *CI* confidence interval, *AUC* area under curve, *IDI* integrated discrimination improvement, *CV* cardiovascular, *RDW* red cell distribution width, *SD* standard deviation

^a^C-statistics and relative IDIs were calculated only in case the parameter was significantly associated with the outcome in Model 2, which was the reference model without the hematological parameter. § None of the C-statistics were significantly higher than that of the baseline model, as well as no relative IDI was statistically significant

**Table 3:** Results of Cox survival analyses for the excess risks associated with hematological parameters, divided into tertiles, for the occurrence of future cardiovascular complications and all-cause mortality

Outcomes Hematological parameters	Model 1 HR (95% CI)	Model 2 HR (95% CI)	C-statistic^a^ (AUC)	Relative IDI^a^ (%)
Total CV events (n = 212)			0.725 (0.684–0.765)	
Haemoglobin	0.79 (0.55–1.15)	0.88 (0.61–1.29)	–	–
RDW	1.06 (0.75–1.48)	1.07 (0.76–1.52)	–	–
Leukocytes	1.16 (0.83–1.63)	0.90 (0.63–1.27)	–	–
Neutrophyls	1.26 (0.89–1.76)	1.08 (0.76–1.53)	–	–
Lymphocytes	0.80 (0.58–1.13)	0.66 (0.46–0.94)‡	0.728§	4.6%§
Monocytes	1.17 (0.83–1.63)	0.93 (0.66–1.33)	–	–
Platelets	1.16 (0.82–1.66)	1.12 (0.78–1.60)	–	–
Neutrophyl-to-lymphocyte ratio	1.55 (1.11–2.16)†	1.47 (1.04–2.07)‡	0.729§	5.4%§
Lymphocyte-to-monocyte ratio	0.79 (0.55–1.13)	0.87 (0.60–1.26)	–	–
Platelet-to-lymphocyte ratio	1.44 (1.03–2.01)‡	1.50 (1.06–2.12)‡	0.729§	4.6%§
Monocyte-to-HDL ratio	1.31 (0.93–1.83)	1.04 (0.72–1.50)	–	–
Major CV events (n = 174)			0.716 (0.673–0.760)	
Haemoglobin	0.74 (0.49–1.10)	0.75 (0.50–1.14)	–	–
RDW	1.07 (0.74–1.55)	1.10 (0.76–1.61)	–	–
Leukocytes	1.30 (0.89–1.90)	1.02 (0.69–1.52)	–	–
Neutrophyls	1.24 (0.85–1.80)	1.08 (0.74–1.59)	–	–
Lymphocytes	0.88 (0.60–1.27)	0.74 (0.50–1.10)	–	–
Monocytes	1.21 (0.83–1.76)	0.96 (0.64–1.43)	–	–
Platelets	1.23 (0.83–1.82)	1.23 (0.83–1.83)	–	–
Neutrophyl-to-lymphocyte ratio	1.32 (0.93–1.88)	1.21 (0.84–1.75)	–	–
Lymphocyte-to-monocyte ratio	0.82 (0.56–1.20)	0.90 (0.61–1.34)	–	–
Platelet-to-lymphocyte ratio	1.40 (0.97–2.02)	1.48 (1.01–2.16)‡	0.721§	6.2%§
Monocyte-to-HDL ratio	1.45 (0.99–2.11)	1.15 (0.76–1.75)	–	–
CV mortality (n = 131)			0.716 (0.668–0.765)	
Haemoglobin	0.83 (0.52–1.32)	0.85 (0.52–1.37)	–	–
RDW	1.43 (0.91–2.22)	1.45 (0.92–2.27)	–	–
Leukocytes	1.24 (0.80–1.91)	0.99 (0.63–1.54)	–	–
Neutrophyls	1.25 (0.81–1.92)	1.11 (0.71–1.72)	–	–
Lymphocytes	0.77 (0.50–1.18)	0.63 (0.40–0.99)‡	0.719§	4.1%§
Monocytes	1.20 (0.78–1.85)	0.94 (0.59–1.48)	–	–
Platelets	0.93 (0.58–1.48)	0.90 (0.56–1.44)	–	–
Neutrophyl-to-lymphocyte ratio	1.39 (0.93–2.07)	1.33 (0.88–2.02)	–	–
Lymphocyte-to-monocyte ratio	0.60 (0.38–0.95)‡	0.62 (0.39–1.00)	–	–
Platelet-to-lymphocyte ratio	1.53 (0.99–2.35)	1.64 (1.06–2.56)‡	0.723§	8.2%§
Monocyte-to-HDL ratio	1.56 (1.00–2.44)‡	1.22 (0.75–1.98)	–	–
All-cause mortality (n = 264)			0.755 (0.717–0.793)	
Haemoglobin	0.70 (0.50–0.98)‡	0.76 (0.54–1.07)	–	–
RDW	1.10 (0.82–1.49)	1.09 (0.80–1.48)	–	–
Leukocytes	1.18 (0.87–1.60)	1.00 (0.73–1.36)	–	–
Neutrophyls	1.17 (0.86–1.60)	1.08 (0.79–1.48)	–	–
Lymphocytes	0.66 (0.49–0.90)†	0.58 (0.42–0.80)*	0.758§	2.6%§
Monocytes	1.37 (1.01–1.86)‡	1.15 (0.83–1.58)	–	–
Platelets	1.11 (0.80–1.52)	1.08 (0.79–1.50)	–	–
Neutrophyl-to-lymphocyte ratio	1.45 (1.08–1.96)‡	1.36 (1.00–1.85)‡	0.756§	0.5%§
Lymphocyte-to-monocyte ratio	0.56 (0.40–0.76)*	0.59 (0.43–0.83)†	0.759§	5.3%§
Platelet-to-lymphocyte ratio	1.39 (1.03–1.87)‡	1.43 (1.05–1.94)‡	0.756§	0.5%§
Monocyte-to-HDL ratio	1.48 (1.09–2.01)‡	1.20 (0.86–1.68)	–	–

Values are hazard ratios of the highest tertile subgroup in relation to the lowest one and their 95% confidence intervals; *p < 0.001; †p < 0.01; ‡p < 0.05

The cut-off values of tertiles of each hematological parameter are the following: haemoglobin 13.0 and 14.2 g/dl; RDW 12.5 and 13.5%; leucocytes 6.3 and 8.1 × 10^3^ cells/mm^3^; neutrophyls 3.5 and 4.7 × 10^3^ cells/mm^3^; lymphocytes 1.8 and 2.5 × 10^3^ cells/mm^3^; monocytes 407 and 552 cells/mm^3^; platelets 210 and 268 × 10^3^ cells/mm^3^; neutrophyl-to-lymphocyte ratio 1.56 and 2.22; lymphocyte-to-monocyte ratio 3.94 and 5.35; platelet-to-lymphocyte ratio 95.68 and 129.78; monocyte-to-HDL ratio 9.37 and 13.83

Model 1 was adjusted for age and sex

Model 2 was adjusted for age, sex, diabetes duration, BMI, smoking, physical activity, office systolic BP, number and classes of anti-hypertensive drugs in use, presence of micro- and macrovascular complications at baseline, baseline HbA_1c_ and LDL-cholesterol, and use of insulin, statins and aspirin

*HR* hazard ratio, *CI* confidence interval, *CV* cardiovascular, *RDW* red cell distribution width

^a^C-statistics and relative IDIs were calculated only in case the parameter was significantly associated with the outcome in Model 2, which was the reference model without the hematological parameter.§ None of the C-statistics were significantly higher than that of the baseline model, as well as no relative IDI was statistically significant

### Risks associated with hematological parameters for incident microvascular complications

Tables 4 (with continuous variables) and 5 (with variables categorized into tertiles) present the adjusted risks of hematological parameters for occurrence of microvascular complications. In general, no hematological parameter was predictive of any microvascular outcome. The exceptions were the hemoglobin concentration (as a continuous variable) for retinopathy outcome, and the lymphocyte-to-monocyte ratio (as a categorical variable) for renal function deterioration. However, none of them significantly improved risk discrimination for these outcomes: hemoglobin increased the C-statistic from 0.750 to 0.752 and the relative IDI demonstrated an improvement of 1.8% for retinopathy risk discrimination; whereas the categorical lymphocyte-to-monocyte ratio increased the C-statistic from 0.680 to 0.693 and the relative IDI showed a statistically-borderline improvement of 14.3% for renal failure risk discrimination. The observed association between hemoglobin concentration and the renal function deterioration outcome was probably due to reverse causality.

**Table 4:** Results of Cox survival analyses for the excess risks associated with continuous hematologic parameters (increments of 1-SD) for the occurrence of future microvascular complications

Outcomes Hematologic parameters	Model 1 HR (95% CI)	Model 2 HR (95% CI)
Retinopathy (n = 161)		
Haemoglobin	0.76 (0.64–0.90)†	0.79 (0.66–0.95)‡
RDW	1.12 (0.97–1.29)	1.15 (0.99–1.35)
Leukocytes	1.13 (0.97–1.32)	1.14 (0.97–1.33)
Neutrophyls	1.06 (0.91–1.25)	1.08 (0.92–1.26)
Lymphocytes	1.10 (0.92–1.31)	1.16 (0.97–1.38)
Monocytes	1.05 (0.91–1.23)	1.00 (0.85–1.18)
Platelets	1.08 (0.91–1.28)	1.08 (0.92–1.27)
Neutrophyl-to-lymphocyte ratio	1.02 (0.87–1.21)	0.98 (0.84–1.14)
Lymphocyte-to-monocyte ratio	0.98 (0.83–1.16)	1.08 (0.91–1.28)
Platelet-to-lymphocyte ratio	0.95 (0.81–1.11)	0.91 (0.77–1.08)
Monocyte-to-HDL ratio	1.06 (0.91–1.23)	1.01 (0.85–1.20)
Renal composite (n = 206)		
Haemoglobin	0.87 (0.74–1.01)	0.87 (0.74–1.02)
RDW	1.02 (0.89–1.17)	1.01 (0.88–1.17)
Leukocytes	1.16 (1.01–1.33)‡	1.12 (0.97–1.29)
Neutrophyls	1.15 (1.00–1.32)‡	1.11 (0.97–1.28)
Lymphocytes	0.98 (0.84–1.16)	0.99 (0.84–1.17)
Monocytes	1.14 (1.00–1.30)	1.11 (0.96–1.27)
Platelets	1.05 (0.91–1.21)	1.06 (0.92–1.23)
Neutrophyl-to-lymphocyte ratio	1.11 (0.97–1.26)	1.07 (0.94–1.23)
Lymphocyte-to-monocyte ratio	0.90 (0.77–1.06)	0.95 (0.81–1.11)
Platelet-to-lymphocyte ratio	0.99 (0.86–1.14)	1.00 (0.86–1.15)
Monocyte-to-HDL ratio	1.15 (1.01–1.32)‡	1.11 (0.96–1.29)
Microalbuminuria (n = 127)		
Haemoglobin	0.89 (0.74–1.08)	0.88 (0.72–1.07)
RDW	1.01 (0.84–1.22)	1.02 (0.85–1.23)
Leukocytes	1.16 (0.98–1.39)	1.15 (0.96–1.38)
Neutrophyls	1.18 (0.99–1.41)	1.16 (0.97–1.40)
Lymphocytes	0.99 (0.80–1.22)	1.02 (0.82–1.26)
Monocytes	1.11 (0.94–1.31)	1.10 (0.92–1.30)
Platelets	1.03 (0.86–1.23)	1.03 (0.86–1.24)
Neutrophyl-to-lymphocyte ratio	1.10 (0.93–1.30)	1.07 (0.90–1.28)
Lymphocyte-to-monocyte ratio	0.90 (0.74–1.10)	0.93 (0.76–1.14)
Platelet-to-lymphocyte ratio	0.97 (0.81–1.16)	0.95 (0.79–1.14)
Monocyte-to-HDL ratio	1.11 (0.93–1.32)	1.10 (0.91–1.32)
Renal failure (n = 104)		
Haemoglobin	0.75 (0.61–0.93)†	0.79 (0.64–0.99)‡
RDW	1.07 (0.89–1.29)	1.06 (0.88–1.28)
Leukocytes	1.15 (0.94–1.41)	1.09 (0.89–1.34)
Neutrophyls	1.15 (0.95–1.40)	1.11 (0.92–1.35)
Lymphocytes	0.94 (0.75–1.19)	0.93 (0.73–1.19)
Monocytes	1.24 (1.03–1.49)‡	1.19 (0.97–1.45)
Platelets	1.03 (0.84–1.28)	1.04 (0.85–1.27)
Neutrophyl-to-lymphocyte ratio	1.15 (0.96–1.38)	1.11 (0.92–1.33)
Lymphocyte-to-monocyte ratio	0.82 (0.64–1.04)	0.86 (0.67–1.11)
Platelet-to-lymphocyte ratio	1.01 (0.83–1.23)	1.02 (0.84–1.24)
Monocyte-to-HDL ratio	1.17 (0.97–1.43)	1.09 (0.88–1.35)
Peripheral neuropathy (n = 179)		
Haemoglobin	0.87 (0.71–1.06)	1.19 (0.93–1.52)
RDW	1.11 (0.92–1.35)	0.85 (0.67–1.08)
Leukocytes	1.16 (0.96–1.40)	1.10 (0.89–1.37)
Neutrophyls	1.19 (0.99–1.44)	1.13 (0.90–1.40)
Lymphocytes	0.91 (0.73–1.14)	0.93 (0.72–1.20)
Monocytes	1.00 (0.83–1.21)	0.99 (0.80–1.23)
Platelets	0.95 (0.78–1.16)	0.93 (0.74–1.17)
Neutrophyl-to-lymphocyte ratio	1.23 (1.02–1.49)‡	1.16 (0.92–1.45)
Lymphocyte-to-monocyte ratio	0.90 (0.74–1.09)	0.90 (0.72–1.12)
Platelet-to-lymphocyte ratio	1.04 (0.86–1.26)	1.00 (0.80–1.25)
Monocyte-to-HDL ratio	1.05 (0.86–1.28)	1.09 (0.86–1.38)

Values are hazard ratios and 95% confidence intervals estimated by Cox analyses for a 1-SD increment in each hematologic parameter; except for peripheral neuropathy endpoint, which is odds ratios and 95% confidence intervals estimated by logistic regressions; †p < 0.01; ‡p < 0.05. The SDs of the hematologic parameters were shown on Table 1

Model 1 adjusted for age and sex

Model 2 was adjusted for age, sex, diabetes duration, BMI, smoking, physical activity, office systolic BP, number and classes of anti-hypertensive drugs in use, presence of micro- and macrovascular complications at baseline, baseline HbA_1c_ and LDL-cholesterol, and use of insulin, statins and aspirin; except for peripheral neuropathy where BMI was substituted by body height and further adjusted for the time interval between baseline and follow-up neuropathy examinations

*HR* hazard ratio, *CI* confidence interval, *RDW* red cell distribution width, *SD* standard deviation

**Table 5:** Results of Cox survival analyses for the excess risks associated with hematologic parameters, divided into tertiles, for the occurrence of future microvascular complications

Outcomes Hematologic parameters	Model 1 HR (95% CI)	Model 2 HR (95% CI)
Rethinopathy (n = 161)		
Haemoglobin	0.57 (0.38–0.86)†	0.65 (0.42–1.00)
RDW	1.40 (0.95–2.07)	1.49 (1.00–2.22)
Leukocytes	1.33 (0.90–1.96)	1.46 (0.97–2.19)
Neutrophyls	1.03 (0.71–1.50)	1.23 (0.83–1.82)
Lymphocytes	1.20 (0.80–1.78)	1.34 (0.89–2.02)
Monocytes	1.19 (0.81–1.75)	1.20 (0.80–1.80)
Platelets	1.00 (0.68–1.48)	1.07 (0.72–1.60)
Neutrophyl-to-lymphocyte ratio	0.97 (0.66–1.43)	0.97 (0.65–1.43)
Lymphocyte-to-monocyte ratio	1.01 (0.68–1.49)	1.18 (0.78–1.76)
Platelet-to-lymphocyte ratio	0.93 (0.63–1.36)	0.84 (0.57–1.24)
Monocyte-to-HDL ratio	1.40 (0.97–2.02)	1.38 (0.91–2.09)
Renal composite (n = 206)		
Haemoglobin	0.67 (0.46–0.98)‡	0.67 (0.45–0.98)‡
RDW	1.13 (0.80–1.58)	1.10 (0.78–1.55)
Leukocytes	1.47 (1.04–2.09)‡	1.38 (0.97–1.98)
Neutrophyls	1.25 (0.90–1.74)	1.19 (0.85–1.67)
Lymphocytes	1.14 (0.81–1.61)	1.18 (0.82–1.69)
Monocytes	1.18 (0.84–1.66)	1.10 (0.77–1.57)
Platelets	1.02 (0.71–1.47)	1.06 (0.74–1.53)
Neutrophyl-to-lymphocyte ratio	1.23 (0.88–1.71)	1.13 (0.80–1.59)
Lymphocyte-to-monocyte ratio	0.75 (0.52–1.07)	0.78 (0.54–1.13)
Platelet-to-lymphocyte ratio	1.04 (0.74–1.45)	1.04 (0.74–1.47)
Monocyte-to-HDL ratio	1.17 (0.84–1.64)	1.04 (0.73–1.49)
Microalbuminuria (n = 127)		
Haemoglobin	0.70 (0.43–1.13)	0.65 (0.40–1.07)
RDW	1.20 (0.77–1.86)	1.22 (0.78–1.91)
Leukocytes	1.55 (0.99–2.43)	1.49 (0.95–2.35)
Neutrophyls	1.43 (0.94–2.18)	1.36 (0.88–2.09)
Lymphocytes	1.09 (0.71–1.69)	1.17 (0.74–1.84)
Monocytes	1.01 (0.66–1.54)	0.99 (0.64–1.53)
Platelets	1.24 (0.77–1.99)	1.22 (0.75–1.97)
Neutrophyl-to-lymphocyte ratio	1.23 (0.81–1.86)	1.12 (0.73–1.72)
Lymphocyte-to-monocyte ratio	0.77 (0.48–1.21)	0.80 (0.50–1.28)
Platelet-to-lymphocyte ratio	1.02 (0.66–1.58)	0.97 (0.62–1.51)
Monocyte-to-HDL ratio	1.00 (0.64–1.55)	0.95 (0.60–1.51)
Renal failure (n = 104)		
Haemoglobin	0.50 (0.29–0.87)‡	0.58 (0.33–1.01)
RDW	1.13 (0.69–1.83)	1.10 (0.68–1.80)
Leukocytes	1.35 (0.83–2.18)	1.19 (0.73–1.95)
Neutrophyls	1.17 (0.74–1.86)	1.11 (0.69–1.78)
Lymphocytes	1.10 (0.68–1.78)	1.03 (0.62–1.71)
Monocytes	1.67 (1.01–2.74)‡	1.50 (0.89–2.53)
Platelets	0.82 (0.50–1.35)	0.87 (0.53–1.45)
Neutrophyl-to-lymphocyte ratio	1.54 (0.95–2.47)	1.47 (0.90–2.41)
Lymphocyte-to-monocyte ratio	0.56 (0.33–0.93)‡	0.58 (0.34–0.99)‡
Platelet-to-lymphocyte ratio	1.13 (0.72–1.79)	1.17 (0.73–1.88)
Monocyte-to-HDL ratio	1.30 (0.82–2.07)	1.09 (0.66–1.81)
Peripheral neuropathy (n = 179)		
Haemoglobin	0.70 (0.43–1.14)	1.45 (0.80–2.64)
RDW	1.56 (0.98–2.48)	0.69 (0.39–1.21)
Leukocytes	1.41 (0.89–2.22)	1.35 (0.80–2.28)
Neutrophyls	1.33 (0.84–2.10)	1.33 (0.78–2.24)
Lymphocytes	0.77 (0.48–1.23)	0.79 (0.46–1.35)
Monocytes	1.02 (0.65–1.61)	0.98 (0.58–1.66)
Platelets	0.92 (0.57–1.48)	0.90 (0.52–1.56)
Neutrophyl-to-lymphocyte ratio	1.54 (0.97–2.46)	1.35 (0.79–2.31)
Lymphocyte-to-monocyte ratio	0.76 (0.47–1.23)	0.75 (0.43–1.30)
Platelet-to-lymphocyte ratio	0.90 (0.57–1.41)	0.92 (0.54–1.54)
Monocyte-to-HDL ratio	1.24 (0.79–1.95)	1.41 (0.82–2.43)

Values are hazard ratios of the highest tertile subgroup in relation to the lowest one and their 95% confidence intervals estimated by Cox analyses; except for peripheral neuropathy endpoint, which are odds ratios and their 95% confidence intervals estimated by logistic regressions. †p < 0.01; ‡p < 0.05. The cut-off values of tertiles of each hematologic parameter were shown on Table 3

Model 1 adjusted for age and sex

Model 2 was adjusted for age, sex, diabetes duration, BMI, smoking, physical activity, office systolic BP, number and classes of anti-hypertensive drugs in use, presence of micro- and macrovascular complications at baseline, baseline HbA_1c_ and LDL-cholesterol, and use of insulin, statins and aspirin; except for peripheral neuropathy where BMI was substituted by body height and further adjusted for the time interval between baseline and follow-up neuropathy examinations

*HR* hazard ratio, *CI* confidence interval, *RDW* red cell distribution width

## Discussion

### Main findings

We investigated in a cohort of middle-aged type 2 diabetic individuals with a long follow-up, the prognostic importance of several hematological parameters for macro- and microvascular outcomes and mortality. This study has three main findings. First, the hematological parameters were mainly predictors of mortality (all-cause and cardiovascular), but more weakly associated with non-fatal CVEs. Higher lymphocytes count was protective and monocytes count was hazardous, and most leukocyte ratios that included lymphocytes were predictive of cardiovascular and all-cause mortality. Second, no hematological parameter was predictive of microvascular complications, except lower hemoglobin concentration for retinopathy and lower lymphocyte-to-monocyte ratio for renal function deterioration. Third, no hematological parameter was able to significantly improve risk discrimination for any outcome. The highest observed relative IDIs were a statistically-borderline 15.5% improvement of monocytes count and monocyte-to-HDL ratio for cardiovascular mortality, and a 14.3% improvement of lymphocyte-to-monocyte ratio for renal failure outcome. Although hematological parameters are easily measurable in routine laboratory exams, they did not appear to add significant prognostic information beyond traditional risk factors in individuals with type 2 diabetes.

### Previous studies on hematologic parameters

As far as we know, there were only 2 previous longitudinal studies that evaluated hematological parameters for adverse outcomes in individuals with diabetes [11, 22, 23]. The first one [11, 22] retrospectively evaluated 338 patients with diabetes and reported that higher NLR was associated with increased odds of developing renal function deterioration (a decrease in eGFR > 12 ml/min to a value < 60 ml/min) over a 3-year period [22], and with higher risks of having a CVE or death over a 4-year period [11]. The second study [23] prospectively followed-up 880 individuals with diabetes over a median of 3.9 years, and reported that higher monocytes count was associated with higher risk of all-cause mortality, which was mainly evident in those with pre-existent macrovascular complications. Our prospective study over a longer follow-up confirmed these initial observations and expanded them by evaluating a larger set of hematological parameters and a comprehensive number of micro- and macrovascular complications outcomes. We demonstrated that, when examined separately, the lymphocytes count was in general the best predictive blood cell count parameter, but the monocytes count was better to predict cardiovascular mortality. The predictive performance of the several white blood cell ratios analyzed appeared to depend basically on the lymphocytes/monocytes count. Total leukocytes, neutrophyls and platelets counts were not predictive of any of the outcomes in isolation; and serum hemoglobin concentration, but not the RDW, was only predictive of retinopathy development/worsening.

Relatively few studies evaluated low absolute lymphocytes count as a prognostic marker [16, 34–37]; three were in patients with cardiovascular diseases [34–36], two in population-based samples [16, 37] and none of them in individuals with diabetes. An observational cohort study showed that low lymphocytes were predictive of all-cause and several cause-specific mortality, including cardiovascular one [37]. The other population-based retrospective study showed that lymphopenia was associated with increased all-cause mortality risk, which was particularly evident in individuals with elevated C-reactive protein and/or RDW [16], a parameter of bone marrow dysregulation that may reflect chronic inflammation [38]. Another investigation performed in a retrospective database of patients who were referred to coronary angiography also demonstrated low lymphocytes count as a predictor of mortality, and the association with higher RDW improved its predictive capacity [37]. In accordance with these studies, we demonstrated in a type 2 diabetes population that low lymphocyte count was a predictor of cardiovascular and all-cause mortalities, and additionally we also showed that low lymphocyte count was a predictor of total CVEs. We may speculate on the reasons why lymphopenia was an independent risk factor for mortality in individuals of different clinical settings. This might be due to a decreased immune vigilance that makes them less capable of surviving to severe disease. As well, reduced lymphocytes could be an indicator of general frailty that confers and an increased risk of any cause of death. Indeed, older age is associated with reduced lymphocytes count [39] that may compromise global immune capacity. Reduced lymphocytes might also indicate chronic inflammatory, metabolic or neuro-endocrine stress factors and thus be associated with reduced survival as an epiphenomenon [16].

### Strengths and limitations

The study has some limitations that shall be noticed. First, it is a prospective observational cohort; hence no causal relationships, nor physiopathological inferences, can be made, but only speculated. Moreover, as with any cohort study, residual confounding due to unmeasured or unknown factors cannot be ruled out. Second, it enrolled mainly middle-aged to elderly individuals with long-standing type 2 diabetes followed-up in a tertiary-care university hospital. Hence, our results might not be generalized to younger individuals with recent onset type 2 diabetes or at primary care management. Otherwise, this study main strength is its well-documented cohort of individuals with type 2 diabetes under standardized care and annual outcomes evaluation over a long 10-year follow-up, which allowed a comprehensive analysis of the excess risks associated with several hematological parameters for separate micro- and macrovascular complications and mortality. And also all individuals who had low or high total leucocytes count had their exam repeated after a month to confirm the results, hence preventing falsely spurious values.

## Conclusions

This prospective long-term cohort study demonstrated that low lymphocytes and high monocytes counts and the leucocytes ratios that mainly included lymphocytes were predictors of cardiovascular events and cardiovascular and all-cause mortalities, and possibly also of renal function deterioration, in individuals with type 2 diabetes. However, their inclusion in the models did not improve risk stratification beyond traditional risk factors. Overall, hematological parameters appeared to add only modest prognostic information in type 2 diabetes.

## Data Availability

The Rio de Janeiro Type 2 Diabetes Cohort Study is an on-going study, and its dataset is not publicly available due to individual privacy of the participants. However, it may be available from the corresponding author on reasonable request.

## Abbreviations

CVE: Cardiovascular event
SBP: Systolic blood pressure
IDI: Integrated discrimination improvement
MACE: Major adverse cardiovascular events
MI: Myocardial infarction
SBP: Systolic blood pressure
NLR: Neutrophyl-to-lymphocyte ratio
RDW: Red blood cell distribution width
